# Discriminating among grounded theory approaches

**DOI:** 10.1111/nin.12261

**Published:** 2018-08-19

**Authors:** Kendra L. Rieger

**Affiliations:** ^1^ Rady Faculty of Health Sciences College of Nursing University of Manitoba Winnipeg Manitoba Canada

**Keywords:** Charmaz, Corbin, Glaser, grounded theory, philosophy of science, qualitative studies, research methodology, Strauss

## Abstract

To rationalize the selection of a research methodology, one must understand its philosophical origins and unique characteristics. This process can be challenging in the landscape of evolving qualitative methodologies. Grounded theory is a research methodology with a distinct history that has resulted in numerous approaches. Although the approaches have key similarities, they also have differing philosophical assumptions that influence the ways in which their methods are understood and implemented. The purpose of this discussion paper is to compare and contrast three widely used grounded theory approaches with key distinguishing characteristics, enabling a more thoughtful selection of approach. This work contributes to the existing literature through contrasting classic Glaserian grounded theory, Straussian grounded theory, and constructivist grounded theory in a systematic manner with prominent distinguishing characteristics developed from a review of the literature. These characteristics included historical development, philosophical perspective, role of the researcher, data analysis procedures, perspective of the grounded theory, and strengths/critique. Based on this analysis, three considerations are proposed to direct the methodological choice for a study: purpose, philosophy, and pragmatics. Understanding the similarities and differences in the grounded theory approaches can facilitate methodological transparency and determine the best fit for one's study and worldview as a researcher.

## 1 INTRODUCTION

When planning a study, the researcher should thoughtfully choose an appropriate methodology based on an awareness of its philosophical underpinnings and its unique characteristics (McEwen & Wills, 2014; Morse, Barrett, Mayan, Olson, & Spiers, 2002; Ryan, 2018). One potential option is grounded theory, a qualitative research methodology that incorporates guidelines for simultaneous data collection and analysis to develop theories about social processes that are grounded in real‐life experiences (Charmaz, 2006; Glaser & Strauss, 1967; McClement & Harlos, 2008; Strauss & Corbin, 1990). Methodologies evolve; they are adapted to fit a changing historical or philosophical milieu (Ralph, Birks, & Chapman, 2015). Grounded theory has a distinct history that has resulted in the development of numerous approaches. Although these approaches have key similarities, they also are based on differing philosophical assumptions that influence the ways in which grounded theory methods are implemented (Charmaz, 2014, 2017). It can be challenging to navigate the complex array of grounded theory approaches. Researchers need a clear understanding of the critical considerations for selecting an approach that best fits their study.

The purpose of this paper is to compare and contrast three widely used grounded theory approaches: classic Glaserian grounded theory (CGGT) (Glaser & Strauss, 1967), Straussian grounded theory (SGT) (Strauss & Corbin, 1990), and constructivist grounded theory (CGT) (Charmaz, 2006). This analysis will illuminate important considerations for choosing a grounded theory approach and facilitate methodological transparency and consistency (Amsteus, 2014; Evans, 2013; Morse et al., 2002). Although this paper builds upon a substantial body of work, this work contributes to the existing literature by contrasting the three approaches in a systematic manner using prominent distinguishing characteristics developed from a review of the literature. In addition, this analysis incorporates important revisions found in Corbin and Strauss's (2015) and Charmaz's (2014) recent editions of their seminal grounded theory texts.

### 1.1 Background

Grounded theory was initially formulated by Glaser and Strauss (1967). Strauss and Glaser actively mentored graduate students, and a number of their students have continued to develop and promote grounded theory. Three of the resultant approaches, CGGT, SGT, and CGT, will be analyzed here as they are the most extensively developed and used (Hood, 2007; Polit & Beck, 2017). Other approaches include situational analysis (Clarke, 2005, 2009), feminist grounded theory (Wuest, 1995), and dimensional analysis (Bowers & Schatzman, 2009; Schatzman, 1991). Situational analysis is often used in combination with CGT, and Clarke (2009) writes, ‘I would not say that situational analysis is a type of grounded theory but rather that it is an extension of grounded theory’ (p. 234).

Viewpoints about the evolution of grounded theory differ. Some scholars view it as the natural maturing of a methodology (McCann & Clark, 2003a) and write of how grounded theory developed within a positivist/postpositivist perspective and that a needed move toward a constructivist perspective has been incorporated (Charmaz, 2006; Mills, Bonner, & Francis, 2006). A positivist paradigm assumes ‘that there is an orderly reality that can be objectively studied’ and that knowledge can be independent of the researcher (Polit & Beck, 2017, p. 739), whereas a postpositivist paradigm assumes that an objective, observable reality exists but acknowledges that it can never be perfectly apprehended as attempts to understand it are influenced by human understanding (Annells, 1996; Ghezeljeh & Emami, 2009; Hall, Griffiths, & McKenna, 2013). A constructivist paradigm assumes that reality cannot be objectively discovered, but instead ‘people, including researchers, construct the realities in which they participate’ (Bryant & Charmaz, 2007a; p. 607). In contrast, Wuest (2012) asserts that grounded theory is firmly rooted in the constructivist paradigm and that researchers bring their own epistemological lens to the study that influences their use of research methods. Others view this evolution as confusing and as an erosion of grounded theory's analytical power (Duchscher & Morgan, 2004; Evans, 2013; Glaser, 2002; Greckhamer & Koro‐Ljungberg, 2005).

The various grounded theory approaches have a recognizable set of ‘family resemblances’ (Bryant & Charmaz, 2007b; p. 11) that are hallmarks of a grounded theory study. Key characteristics (see Table 1) include that grounded theory elucidates a process; begins with inductive logic; encompasses simultaneous data collection, analysis, and theory construction; incorporates constant comparison and memo writing; employs theoretical sampling; and focuses on the generation of a grounded theory (Charmaz, 2006, 2014, 2017; Corbin, 2009; Corbin & Strauss, 2015; Duchscher & Morgan, 2004; Elliot & Lazenbatt, 2005; Glaser & Strauss, 1967; Hall & Callery, 2001; Hood, 2007; Hunter, Murphy, Grealish, Casey, & Keady, 2011; McCrae & Purssell, 2016; Mills et al., 2006; Morse, 2001; Ralph et al., 2015; Walker & Myrick, 2006). Although there are identifiable grounded theory characteristics, there are also noteworthy differences between the approaches that need to be examined before embarking on a study (Charmaz, 2014; Mills et al., 2006).

**Table 1:** Similarities between GT approaches informed by the literature

Characteristic	Description
Elucidates a process	Explicating a social process (Hood, 2007; Morse, 2001; Udod & Racine, 2017), which is comprised of ‘unfolding temporal sequences that may have identifiable markers with clear beginnings and endings and benchmarks in between’ (Charmaz, 2014, p. 17).
Begins with inductive logic	Starting by looking at the data with no ideas to prove or disprove. Issues of importance emerge from people's descriptions (Corbin, 2009; Hunter et al., 2011; Mills et al., 2006).
Simultaneous data collection, analysis, and theory construction	In concurrent data collection and analysis, analysis begins early with the first few interviews and focuses on developing theoretical ideas (Charmaz, 2006; Elliot & Lazenbatt, 2005; Hall & Callery, 2001).
Constant comparison	Developing successively more abstract ideas through comparing data with data, data with code, code with code, code with category, and category with category, in order to identify commonalities and differences (Charmaz, 2014; Corbin, 2009; Duchscher & Morgan, 2004; Elliot & Lazenbatt, 2005; Hall & Callery, 2001; Hunter et al., 2011; Walker & Myrick, 2006).
Memo writing	Keeping a written record of comparisons and analytical thoughts about the data/data analysis process in order to develop theoretical ideas and direct theoretical sampling (Corbin, 2009; Duchscher & Morgan, 2004; Elliot & Lazenbatt, 2005; Hood, 2007).
Theoretical sampling	As theoretical ideas are developed, concepts derived from the early analysis will guide the collection of additional data to elaborate the developing theoretical categories and address conceptual gaps. Researchers often start with convenience/purposive sampling and then move to theoretical sampling. Theoretical saturation, the point at which new data no longer provide theoretical insights, is the criteria for stopping data collection (Charmaz, 2006; Corbin, 2009; Hunter et al., 2011; McCrae & Purssell, 2016; Walker & Myrick, 2006).
Generation of a grounded theory	Developing theoretical abstractions that are grounded in the data and encompass the variation of participants’ experiences. Most grounded theories are substantive theories—they explicate delimited phenomenon in a particular area. When theoretical ideas transfer across areas, they can be developed into a more formal theory encompassing a higher level of abstraction with broader applicability (Charmaz, 2014; Corbin, 2009; Glaser & Strauss, 1967; Morse, 2001; Streubert & Carpenter, 2011).

### 1.2 Data sources

This discussion paper is based on literature ranging from 1965 to 2017. CINAHL, PsycINFO, and MEDLINE were searched from their respective inception dates until 2017 to find relevant literature, using various combinations of the following keywords: ‘grounded theory,’ ‘Charmaz,’ ‘Strauss,’ ‘Corbin,’ and ‘Glaser.’ Additional materials were drawn from library catalogs, reference lists of relevant work, and the gray literature.

## 2 DISTINGUISHING CHARACTERISTICS FOR CONTRASTING THE APPROACHES

This analysis will examine six distinguishing characteristics that were identified in the literature as constituting the most significant differences between approaches and being the most essential considerations for deciding on a grounded theory approach. First, each approach is rooted in a different historical context (Clarke, 2009; Greckhamer & Koro‐Ljungberg, 2005; Ralph et al., 2015). Second, that historical context was often associated with a particular philosophical tradition that likely influenced the modifications made to CGGT. Although all three approaches have roots in symbolic interactionism (Corbin & Strauss, 2008; McCrae & Purssell, 2016), they are also imbued with either positivist or constructivist underpinnings that affect research practices (Charmaz, 2000, 2017; Ralph et al., 2015). Third, as a result of divergent philosophical assumptions, the role of the researcher differs between the various approaches (Charmaz, 2006). The researcher's role involves ideas about the nature of the research–participant relationship (Heath & Cowley, 2004), theoretical sensitivity (Mills et al., 2006), treatment of the literature (Mills et al., 2006), and identification of research questions (Hunter et al., 2011). Fourth, differences in the views of data and data analysis procedures are significant, as is the emphasis on inductive, deductive, or abductive logic (Charmaz, 2009; Heath & Cowley, 2004; Kelle, 2005; Reichertz, 2010; Walker & Myrick, 2006). Fifth, divergent perspectives also exist of the grounded theory that emerges from the analysis (Heath & Cowley, 2004; Mills et al., 2006). Sixth, these cumulative differences contribute to unique methodological strengths and limitations (Polit & Beck, 2012).

## 3 CONTRASTING DIFFERENCES: DISTINGUISHING CHARACTERISTICS OF THE THREE APPROACHES

### 3.1 Classic Glaserian grounded theory

#### 3.1.1 History and origins

Glaser and Strauss (1967) published their revolutionary grounded theory text at the beginning of the qualitative revolution during an era when postpositivism dominated research thinking (Charmaz, 2000, 2006). Their different backgrounds influenced much of their methodological development. Strauss was an expert in symbolic interactionism and Glaser was a quantitative researcher (Stern, 2009b). Glaser and Strauss’ goal was to establish qualitative research as a rigorous process with reliable data analysis and defend grounded theory from positivist critique (Charmaz, 2006). These influencing factors combined to create a rigorous methodological process that had a positivist direction (Bryant & Charmaz, 2007c). Their differences also ‘planted the seeds of divergent directions’ (Charmaz, 2009; p. 129) which eventually led to the demise of their research relationship. Glaser argued that CGGT is the pure form of grounded theory. Although he claims to have stayed true to the original method, Stern (2009a) notes that there has been a subtle evolution of ideas in Glaser's writing. He has continued to develop CGGT in his subsequent publications (Glaser, 1978, 1992, 2001, 2002, 2003, 2005, 2007, 2009, 2011, 2012, 2014a,b,c, 2015, 2016a,b) by, for example, explaining abstract concepts such as theoretical sensitivity (Glaser, 1978), developing numerous theoretical coding families (Glaser, 1978, 2005), and describing in detail how to conceptualize data (Artinian, 2009; Glaser, 2001, 2003, 2005). Glaser also launched the Grounded Theory Institute and the associated journal, *Grounded Theory Review: An International Journal*, to continue promoting, developing, and refining CGGT (Kenny & Fourie, 2014).

#### 3.1.2 Philosophical perspective

The seminal CGGT texts have little discussion of underlying philosophical assumptions (Bryant, 2009), but these assumptions can be inferred and seem to echo postpositivist presuppositions. Some researchers assert that CGGT is based on a positivist realist ontology that assumes an orderly real world exists which can be objectively observed (Charmaz, 2000; Clarke, 2005), while others contend that CGGT is actually based on a postpositivist critical realist ontology that assumes a real world exists but acknowledges that it is impossible for people to truly perceive it (Annells, 1996; Hall et al., 2013; Ralph et al., 2015). Further, Charmaz (2000) argues that CGGT is based on an objectivist epistemology which assumes that the researcher is separate from what is being studied and aims for unbiased discovery of new knowledge. Charmaz (2000) described a continuum between objectivist and CGT, and Mills et al. (2006) proposed that a methodological spiral is present between postpositivist and constructivist approaches. Both placed CGGT as the most positivist/postpositivist on these spectrums. The title *The Discovery of Grounded Theory* (Glaser & Strauss, 1967) conveys the assumption that an external reality exists which can be found (Charmaz, 2000), and CGGT researchers assert that the data reveal a true theory (Mills et al., 2006). Other CGGT characteristics consistent with an objectivist epistemology include valuing a neutral expert observer, separating data and the observer, and developing parsimonious abstractions free of context (Glaser & Strauss, 1967; Mills et al., 2006).

#### 3.1.3 Role of the researcher

Consistent with the tenets of objectivism, CGGT researchers should have as few predetermined thoughts as possible and maintain their role as detached observers (Glaser & Strauss, 1967; Hall & Callery, 2001). Glaser (2002) wrote that through constant comparison, ‘Personal input by a researcher soon drops out as eccentric and the data become objectivist not constructionist’ (p. 6). The focus is on the neutralization of researcher bias through constant comparison, instead of on reflexivity (Charmaz, 2017; Mruck & Mey, 2007). Further, *theoretical sensitivity* is ‘the ability to recognize and extract from the data elements that have relevance for the emerging theory’ (Birks & Mills, 2011, p. 176), and in CGGT, it is achieved through immersion in the data (Glaser, 1978). Although Glaser's coding families can enhance theoretical sensitivity (Table 2), these codes are applied later in the analysis (Glaser, 1978; Walker & Myrick, 2006). In CGGT, the literature review is delayed to prevent the researcher from developing preconceived ideas, and the researcher does not need an initial research question or guiding theory because those elements could foster unwanted researcher influence (Glaser & Holton, 2007). However, Glaser (1978) does endorse the use of emergent fit within grounded theory analysis to see whether a pre‐existing category from a previous study fits with the emerging data in the current study (Artinian, 2009; Wuest, 2000). Emergent fit involves an iterative process of constant comparison with the existing literature and modification of the relevant concepts so that only elements of the pre‐existing theory that fit the data will remain (Glaser, 1978; Wuest, 2000).

**Table 2:** Data analysis procedures

Grounded theory approach	Classic Glaserian grounded theory (Glaser, 1978, 1992; Glaser & Strauss, 1967)	Straussian grounded theory (Corbin & Strauss, 2008, 2015; Strauss & Corbin, 1990, 1998)	Constructivist grounded theory (Charmaz, 2006, 2014)
Coding stages	Substantive coding Open coding Coding through immersion in the data This subphase ends with the discovery of the core category Selective coding Selectively coding data that relate to the core category Theoretical coding Integrating substantive codes into a grounded theory A theoretical coding family may be used	Open coding Coding pieces of data with line‐by‐line coding. Identifying categories, and their properties or dimensions Axial coding Putting the fractured data back together by making connections between categories and subcategories Involves the use of a coding paradigm to identify these links Selective coding Selecting a core, or central, category Conceptually relating all categories to the core category, and to the other categories in order to form the grounded theory In the 2008 and 2015 texts, selective coding is not used and this final process is referred to as theoretical integration	Initial coding Studying fragments of data and labeling them with codes Focused coding Using initial codes that reappear frequently, and are the most relevant, to theoretically code all future data
Analytical tools used during data analysis	Theoretical coding families Choosing a family of theoretical codes to reintegrate the fractured data There are at least 18 theoretical coding families, which are flexible sets of codes derived primarily from sociological theory	The coding paradigm The coding paradigm came from one of Glaser's coding families (the six C's) and is used during axial coding or to code around a category Focuses the researcher on the conditions of the phenomenon, actions/interactions and emotions of participants, and consequences of the actions/interactions or emotional responses Conditional/consequential matrix A coding device to make connections between the macro and micro conditions affecting the phenomenon of study Used during axial coding or selective coding Exemplars of other analytic tools for probing the data Flip‐flop technique: turning a concept inside out by looking at opposite extreme conception of a concept to highlight its properties Waving the red flag: when words such as ‘never’ or ‘always’ arise, this occurrence should alert the researcher to investigate this claim further	Potential tools for analysis Can employ analytical tools developed by other grounded theorists in a CGT study if they are appropriate for the emerging analysis

#### 3.1.4 Data and data analysis

Through claims that data are objective and discovered (Glaser, 2002), data become a true representation of the research participants’ realities in CGGT. The contexts of the data are not considered unless it emerges as a code (Glaser & Holton, 2007). The goal of CGGT analysis is to watch objectively for the emergence of the grounded theory through data analysis procedures (Glaser & Strauss, 1967).

Coding involves two phases: substantive coding, which incorporates two subphases of open and selective coding, produces categories and their properties, and identifies a core category; and theoretical coding, which occurs at the conceptual level, involves the use of coding families in some studies, and weaves the categories into a grounded theory (Glaser & Strauss, 1967) (Table 2). Key differences between grounded theory approaches are apparent in coding—specifically, in the timing and level of researcher intervention (Evans, 2013; Walker & Myrick, 2006). These differences have fueled a debate about allowing data to emerge versus forcing the data with a preconceived framework, with Glaser claiming that CGGT alone allows theory to emerge because it has no external frame until theoretical coding is conducted (Glaser, 1978; Heath & Cowley, 2004). Deduction and verification of hypotheses should be left for quantitative researchers, as can be seen when Glaser and Strauss (1967) wrote, ‘Comparative analysis both subsumes and assumes verification and accurate description, but only to the extent that the latter are in the services of generation’ (p. 28). Although they view constant comparison as incorporating verification, it is only used within the primary processes of induction and generation of theoretical ideas.

#### 3.1.5 The grounded theory

In CGGT, views of the grounded theory have positivist leanings (Charmaz, 2006), and Glaser (2002) stated that CGGT ‘makes the generated theory as objective as humanly possible’ (p. 5). The theory represents a correspondence with reality (Thomas & James, 2006), and a certain set of data should produce the same grounded theory if the research was rigorous (Duchscher & Morgan, 2004; Heath & Cowley, 2004). Glaser (1992) also asserted that the grounded theory should have explanatory power. In CGGT, the grounded theory surrounds a core category, or major theme, that unites all of the conceptual categories (Glaser, 1978). In particular, each grounded theory approach offers distinct evaluation criteria (Table 3).

**Table 3:** Evaluation criteria for grounded theory studies

Glaserian classic grounded theory (Glaser, 1978)	Straussian grounded theory (Corbin & Strauss, 2008, 2015)	Constructivist grounded theory (Charmaz, 2006, 2014)
1. Fit 2. Work 3. Relevance 4. Modifiability	In their 2008 text, Corbin and Strauss provided criteria for judging the quality of research findings: 1. Fit 2. Applicability 3. Concepts 4. Contextualization of concepts 5. Logic 6. Depth 7. Variation 8. Creativity 9. Sensitivity 10. Evidence of memos Corbin and Strauss (2008) also provided 13 criterion in question form for judging the credibility of the research (pp. 307–309). For example, for criterion one the reviewer would ask, ‘How was the original sample selected? How did later sampling occur?’ (p. 307). In their revised 2015 text, Corbin and Strauss proposed 16 components to evaluate the methodological consistency of a grounded theory study in question form, and 17 components of quality and applicability in question form (pp. 350–351). For example, for the first quality/applicability component the reviewer would ask, ‘What is the core category, and how do the major categories relate to it? Is there a diagram depicting these relationships?’ (p. 351).	1. Credibility 2. Originality 3. Resonance 4. Usefulness

#### 3.1.6 Strengths and critique

What Glaser and Strauss (1967) proposed was revolutionary and a powerful force for the legitimization of qualitative research (Charmaz, 2009). CGGT is a flexible approach that is less prescriptive than SGT (Evans, 2013), yet it is still rigorous and results in a high level of abstraction. Because CGGT researchers bring few preconceived notions, some assert, the approach results in greater theoretical completeness (Evans, 2013; Heath & Cowley, 2004). Nevertheless, CGGT has come under significant critique for its lack of explicit discussion of the philosophical assumptions that underlie it (Greckhamer & Koro‐Ljungberg, 2005). Numerous scholars have argued that the concept of the uncontaminated researcher is a naïve notion that is not congruent with the tenets of qualitative research (Charmaz, 2008b; Corbin, 1998; Kelle, 2005; Mills et al., 2006). Other critiques include that CGGT has a limited ability to explicate a meaningful understanding due to a reliance on participants’ overt concerns (Charmaz, 2000), an overuse of sociological terms and laissez‐faire guidelines (Charmaz, 2000; McCann & Clark, 2003a), and a tendency to privilege the researcher's knowledge by valuing a distance between the researcher and participants. In the absence of strategies to address this power differential, researchers can elevate their own assumptions and interpretations to an objective status (Bryant & Charmaz, 2007c; Hall & Callery, 2001).

### 3.2 Straussian grounded theory

#### 3.2.1 History and origins

The differences between Glaser and Strauss were stressed when the partnership of Strauss and Juliet Corbin emerged (Morse, 2009) and they published the *Basics of Qualitative Research* (Strauss & Corbin, 1990). Their goal was to offer an accessible grounded theory text with well‐described techniques (Heath & Cowley, 2004). However, this book caused a rift between Glaser and Strauss, and Glaser (1992) wrote a scathing critique of SGT's prescribed approach. By the 1990s, two divergent grounded theory approaches were evident: CGGT (Glaser & Strauss, 1967), and SGT (Strauss & Corbin, 1990, 1998), which seems less positivistic/postpositivistic (Bryant & Charmaz, 2007c). Strauss died in 1996; however, Corbin continued to develop their approach and believes she has stayed true to the key aspects of their earlier work (Corbin & Strauss, 2008, 2015). Regardless of the significant revisions found in the third and fourth editions (Corbin & Strauss, 2008, 2015), many researchers continue to use earlier versions of the SGT approach (Charmaz, 2014).

#### 3.2.2 Philosophical perspective

The philosophical perspective of SGT has pragmatist and symbolic interactionist foundations, but it is also somewhat ambiguous and has evolved over time (Corbin & Strauss, 2008; Greckhamer & Koro‐Ljungberg, 2005). Symbolic interactionism is an interpretive theoretical perspective ‘derived from pragmatism which assumes that people construct selves, society, and reality through interaction’ (Charmaz, 2014; p. 344). Symbolic interactionists do not deny that there is a reality, but assert that it is socially interpreted, and that understanding these constructions is important to comprehend human behavior (Charon, 2010). Corbin (1998) wrote about their first book (Strauss & Corbin, 1990): ‘It was meant as a supplemental text, therefore it omits most of the epistemological foundations’ (p. 122). Numerous scholars interpret SGT as moving toward constructivism in the first two texts (Annells, 1997; Lomborg & Kirkevold, 2003; McCann & Clark, 2003a; Mills et al., 2006). Evidence for constructivist leanings can be found in statements like ‘theorizing is the act of constructing’ (Strauss & Corbin, 1998, p. 25) and ‘there are many alternative interpretations of data’ (Corbin, 1998; p. 122). However, others argued that the original version of SGT is positivist, uses linear approaches, and has a realist ontology, because it assumes an external reality, and objectivist epistemology, because it values unbiased data collection (Charmaz, 2000; Evans, 2013; Hall & Callery, 2001). This vacillation between positivism and constructivism can also be seen as a ‘struggle to move within the changing moments of qualitative research’ (Mills et al., 2006, p. 4).

An evolution of philosophical perspectives has occurred within SGT (Ralph et al., 2015). In 1994, Strauss and Corbin started positioning themselves as relativist pragmatists, and in the third and fourth texts, Corbin claimed to be a constructivist (Corbin & Strauss, 2008, 2015). Although she admitted their early work was more positivistic, she noted that her new views are reflected in recent work through the acknowledgement of the existence of multiple complex realities that relate to real events, the varied participant responses to events, and the construction of theories by researchers (Corbin & Strauss, 2008, 2015). As well, research techniques and procedures are framed as tools instead of directives. Charmaz (2014) also viewed SGT as moving toward constructivism in the third edition of Corbin and Strauss's work (2008).

#### 3.2.3 Role of the researcher

In contrast to CGGT, in SGT the researcher is not viewed as a blank slate and has an interpretive role (Corbin & Strauss, 2015), which is more consistent with the stance of constructivism (McCann & Clark, 2003a,b). The researcher's experience can enhance theoretical sensitivity, facilitate the generation of hypotheses, and create a base for making comparisons (Corbin, 1998). In a later edition of their text, Corbin and Strauss (2008) wrote that ‘objectivity in qualitative research is a myth’ (p. 32). Throughout the evolution of SGT, reflexivity is increasingly acknowledged as vital to ensuring that the researcher's perspectives are helpful, rather than restrictive, during data collection and analysis (Corbin & Strauss, 2008, 2015). Strauss and Corbin (1990) asserted that theoretical sensitivity is enhanced not just through immersion in the data, as with CGGT, but also through the use of analytic tools such as the flip‐flop technique and waving the red flag (Table 2). In contrast to CGGT, in SGT the literature can inform research questions, increase theoretical sensitivity, and stimulate reflections (Strauss & Corbin, 1998). Furthermore, a broad research question should be stated before the research begins (Strauss & Corbin, 1990). Similar to CGGT, a SGT researcher should not normally begin their research with an extant theory, although later on in the analysis a theoretical framework can be a useful lens through which to view the data (Corbin & Strauss, 2015).

#### 3.2.4 Data and data analysis

Given that data are not viewed as being completely separate from the researcher, SGT researchers acknowledge that ‘there is no one “reality” out there waiting to be discovered’ (Corbin & Strauss, 2008; p. 10). In contrast to CGGT and CGT, in SGT data analysis the focus is on using analytical tools, which are thinking techniques that promote interaction between the researcher and the data, and this approach is the only one that originally had three distinct phases of coding: open, axial, and selective (Walker & Myrick, 2006) (Table 2). These phases are more complex and detailed than in CGGT or CGT, and the researcher intervenes more intensively using analytical tools and questions (Strauss & Corbin, 1998; Walker & Myrick, 2006). Open coding involves coding the text, and discovering a category's properties and dimensionalizing them (Strauss & Corbin, 1998). Axial coding involves putting fractured data back together with the use of the coding paradigm, a framework of questions derived from one of Glaser's coding families that facilitates the identification of the relationship between structure and process and the linking of categories and subcategories (Corbin & Strauss, 2015). The researcher groups code into three components: conditions, inter/actions and emotions, and consequences (Corbin & Strauss, 2008; Thompson, McClement, & Daeninck, 2006). At last, a core category is identified during selective coding, and the researcher conceptually relates all categories to the core, or central, category (Strauss & Corbin, 1990). In Corbin and Strauss's (2008, 2015) latest two texts, open coding and axial coding are presented as iterative and flexible processes, the term *selective coding* is not used, and this final process is referred to as *theoretical integration*. The authors have also developed a conditional/consequential matrix to make connections between the macro and micro conditions (Corbin & Strauss, 2008).

The SGT researcher intervenes earlier in the analysis process with the use of a preconceived coding paradigm during axial coding and before the development of a core category, differing from CGGT, in which flexible theoretical codes are used after a core category is identified (Walker & Myrick, 2006). Another key distinction is the emphasis on deduction and verification of hypotheses, as opposed to induction, as in CGGT (Heath & Cowley, 2004; McCann & Clark, 2003a). Strauss and Corbin (1990, 1998) contended that researchers make statements about the relationship between concepts, and these hypotheses are then validated in subsequent data collection. In later texts, there is less emphasis on verification and more on the interplay of induction and deduction (Corbin & Strauss, 2008; Walker & Myrick, 2006).

#### 3.2.5 The grounded theory

In SGT, the view of the grounded theory has both positivist and interpretivist elements (Charmaz, 2006). Consistent with positivist definitions of theory (Charmaz, 2006), Corbin and Strauss (2008) defined theory as ‘a set of well‐developed categories (themes, concepts) that are systemically interrelated through statements of relationship to form a theoretical relationship that can be used to explain some phenomena’ (p. 55). However, the grounded theory is viewed as one possible interpretation that does not exactly represent reality (Corbin & Strauss, 2015). Similar to CGGT researchers, SGT researchers aim to identify a core category for the grounded theory (Corbin & Strauss, 2015).

#### 3.2.6 Strengths and critique

Strengths of SGT include that the approach offers a clear description of its complex research procedures (Walker & Myrick, 2006) and that SGT enables the researcher to focus on both the micro and macro conditions using the conditional/consequential matrix (Mills et al., 2006; Walker & Myrick, 2006). Some scholars assert that the rigorous analytic tools, such as the coding paradigm, enable the construction of sufficiently analytical theories (McCann & Clark, 2003c; Mills et al., 2006). However, others argue that SGT is rigid and focuses on systematic procedures that interfere with the researchers’ sensitivity to the data and promote a power differential between researcher and participants (Bryant & Charmaz, 2007c; Evans, 2013; Heath & Cowley, 2004; Hunter et al., 2011). The early timing and intensity of researcher intervention and the emphasis on using analytical tools can force data into preconceived ideas instead of allowing the grounded theory to emerge (Charmaz, 2000; Glaser, 1992; Walker & Myrick, 2006).

### 3.3 Constructivist grounded theory

#### 3.3.1 History and origins

Kathy Charmaz, a sociologist, developed CGT based on ideas from two of her mentors: Barney Glaser and Anselm Strauss (Charmaz, 2017; Morse, 2009). CGT returns to CGGT strategies but has repositioned grounded theory, shifting it from its positivist underpinnings to those of constructivism (Bryant & Charmaz, 2007c; Charmaz, 2017). Charmaz (1995) began writing about her ideas during the ‘crisis of representation’ (Denzin & Lincoln, 2005, p. 18), which was a period when qualitative researchers were seeking new models of truth and when objectivity became problematic (Birks & Mills, 2011). Charmaz (2008a) claimed that CGT ‘builds on Glaser's useful methodological strategies … but it does not duplicate the logic of inquiry in classic grounded theory statements’ (p. 136).

#### 3.3.2 Philosophical perspective

In contrast to CGGT and SGT theorists, Charmaz explicitly took a constructivist stance with CGT's relativist ontology and subjective epistemology (Charmaz, 2006, 2014; Mills et al., 2006). Constructivist grounded theorists acknowledge that reality is a social construction (Ghezeljeh & Emami, 2009). CGT researchers do not deny the existence of objectively true worlds, but they are more concerned with the ‘world made real in the minds and through the words and actions of its members’ (Charmaz, 2000; p. 523). CGT has a subjective epistemology in that it assumes that researchers are not separate from the research and that knowledge is cocreated (Charmaz, 2000). Both Charmaz (2000) and Mills et al. (2006) positioned CGT on the far end of the positivist‐constructivist continuum compared with CGGT and SGT.

#### 3.3.3 Role of the researcher

In contrast to CGGT, the CGT researcher is a cocreator of knowledge (Charmaz, 2000). As a result, the researcher influences the research through interactions with participants and data (Mulugeta, Williamson, Monks, Hack, & Beaver, 2017), and the researcher's experience is valued in this process (Charmaz, 2014). Charmaz (2014, 2017) also asserted that researchers need to be reflexive to avoid forcing their preconceived ideas on the data, and she viewed memo writing as key to reflexivity. In CGT, theoretical sensitivity or sensitizing concepts can guide the research, but they are only ‘points of departure’ from which to develop ideas (Charmaz, 2014, p. 30). Also, in contrast to CGGT, the use of extant theories can develop theoretical sensitivity, a literature search orients the researcher, and a research question is developed before the research commences (Charmaz, 2014; Harling & Turner, 2012).

#### 3.3.4 Data and data analysis

Constructivist grounded theory researchers acknowledge the subjectivity of data and data analysis, a stance that aligns CGT with constructivism. The researcher and participants coconstruct the data, and thus, data are a product of the research instead of an observation or a window on reality (Charmaz, 2006, 2014). Charmaz (2014) emphasized using intensive interviewing to attend to participants’ stories and to construct theories. In contrast to those who espouse CGGT, CGT researchers use thoughtful probes to understand implicit meanings and are attentive to the participants’ context (Charmaz, 2014). In contrast to SGT, CGT analysis focuses on flexible data analysis procedures to elucidate social processes, instead of on the application of external devices (Charmaz, 2006).

Constructivist grounded theory data analysis is similar to that of CGGT in that it includes at least two phases, initial and focused coding, and has less researcher intervention than SGT (Charmaz, 2014; Mills et al., 2006) (Table 2). Initial coding involves labeling data with codes. Focused coding uses initial codes that reappear frequently, and are the most relevant, to code and categorize larger portions of data. Focused codes are more conceptual, and certain focused codes are elevated to abstract categories (Charmaz, 2006; Ripat & Woodgate, 2012). Theoretical integration begins with focused coding and culminates in the final grounded theory (Charmaz, 2014). External frameworks such as axial coding (Strauss & Corbin, 1998) and theoretical codes (Glaser, 1978) can be useful if they ‘earn their way’ into the analysis (Charmaz, 2014, p. 153).

Constructivist grounded theory is the first grounded theory approach to clearly describe *inductive‐abductive logic*, which is the iterative process of the researcher moving back and forth between data and conceptualization, as a key part of data analysis (Charmaz, 2009). *Abductive logic* involves contemplating possible theoretical explanations for the researcher's initial observations, and then attempting to confirm or disconfirm these tentative ideas to arrive at the most plausible explanation (Charmaz, 2014). Grounded theory researchers make inferences from the data (inductive) and then check them using theoretical sampling and additional data (abductive) (Charmaz, 2014). Abductive logic is situated between inductive logic, the focus of CGGT, and deductive logic (Clarke, 2009). Some assert that certain SGT texts describe the use of abductive logic without using the term (Bryant, 2009; Reichertz, 2010).

#### 3.3.5 The grounded theory

Charmaz (2014) proposes a continuum from positivist theory, which aims to explain and predict through ‘explanatory generalizations that theorize causation,’ to interpretivist theory, which is emphasized in CGT and aims for ‘abstract understandings that theorize relationships between concepts’ (p. 228). An interpretivist theory makes sense of the studied phenomenon by conceptualizing it in abstract terms but does not focus on explaining causality. Further, in CGT, the theoretical understanding emerges from the researchers’ interaction with the data rather than objectively from the data for an unbiased researcher to discover. The grounded theory ‘constructs an image of a reality, not the reality’ (Charmaz, 2000; p. 523), and there are likely many possible theoretical interpretations from one set of data (Greckhamer & Koro‐Ljungberg, 2005). In addition, the grounded theory may be comprised of theoretical concepts with no core category, given that CGT emphasizes multiple realities that may lack a unitary theme (Charmaz, 2006; Heath & Cowley, 2004).

#### 3.3.6 Strengths and critique

Constructivist grounded theory offers a reinterpretation of grounded theory, thereby enabling researchers to employ grounded theory without embracing positivist assumptions (Charmaz, 2000, 2017). Constructivist grounded theorists seek to develop mutuality with participants, which uncovers hidden meanings and results in an insightful theoretical interpretation (Charmaz, 2006). CGT also addresses the tension between objective emergence from the data, as in CGGT, and the researcher forcing the data with her or his intervention, as in SGT, by focusing on researcher reflexivity and abductive reasoning (Bryant, 2009; Charmaz, 2017; Duchscher & Morgan, 2004; Kelle, 2005). CGT offers more clearly described strategies than does CGGT, but it offers fewer prescriptive procedures than SGT (Charmaz, 2000). In contrast, Glaser (2002) argued that CGT erodes the analytical power of grounded theory and claimed that CGT forces the data through interview guides and prolonged interviews, remodels CGGT into a form of qualitative data analysis, and neglects using careful CGGT strategies to render the data objective. Others question whether a methodology can be radically changed and still be the same methodology (Breckenridge, Jones, Elliott, & Nicol, 2012; Greckhamer & Koro‐Ljungberg, 2005; Morse, 2009) and whether Charmaz is trying to legitimize her work by associating it with grounded theory (Thomas & James, 2006).

## 4 IMPLICATIONS FOR NURSING: SELECTING A GROUNDED THEORY APPROACH

With all the available grounded theory approaches, how can a discerning researcher decide on the best approach for their study? I propose three considerations which flow from the distinguishing characteristics analyzed to direct one's methodological choice for a grounded theory study: purpose, philosophy, and pragmatics.

Of utmost importance is that the grounded theory approach must be congruent with the desired knowledge and the study's purpose (Corbin & Strauss, 2008; Crotty, 1998; Evans, 2013; Houghton, Hunter, & Meskell, 2012). For example, if the researcher's goal is to discover an explanatory theory and identify variables, then CGGT may be a more appropriate choice. However, if a researcher desires a theoretical understanding of a situated process, then this type of knowledge may be better elicited with CGT (Charmaz, 2014; Evans, 2013). This deliberation ensures that the type of theory desired from the study is consistent with the methodological approach selected (Mills et al., 2006).

It is also crucial to consider one's philosophical beliefs about the form of reality (ontology) and the nature of knowledge development (epistemology). Numerous scholars discuss the importance of choosing a research approach that is congruent with the researcher's personal beliefs (Corbin & Strauss, 2008; Crotty, 1998; Evans, 2013). I argue that CGGT is imbued with positivist/postpositivist assumptions, that SGT has evolved from a postpositivist position toward a constructivist view, and that CGT is clearly based on constructivist ideas about reality and knowledge development. One must determine the best fit with their personal philosophical beliefs and this process necessitates thoughtful reflection (Howard‐Payne, 2016).

As can be seen from this analysis, philosophical foundations have significant implications for the methods used in a grounded theory study. For example, the role of the researcher varies between approaches. Researchers need to consider whether they aim to take an objective role to watch for the emergence of concepts that reside in the data (CGGT) or believe that they should acknowledge and reflectively incorporate their previous knowledge and experiences into study procedures (SGT and CGT) (Breckenridge et al., 2012; Charmaz, 2014; Howard‐Payne, 2016). The same holds true for data analysis. SGT's use of axial coding with a coding paradigm is intended to help researchers develop sufficiently theoretical ideas; however, some researchers may value CGGT or CGT for their focus on the emergence of ideas from the data unencumbered by a preconceived framework (Artinian, 2009; Charmaz, 2006). At last, it is critical to consider how one intends to use extant theories. While CGT encourages the use of theory from the outset of the study, CGGT and SGT recognize the value of extant theory once data analysis begins. Although some researchers use a selected grounded theory approach without fully realizing the implications on study methods, consistency between the methodological approach and its associated methods is essential in rigorous research (Corbin & Strauss, 2015; Morse et al., 2002). Grounded theory approaches have been rigorously developed over the years, and researchers should use them in a manner consistent with their stated procedures to produce credible findings (Corbin & Strauss, 2015).

There are pragmatic considerations that also influence the researcher's selection of a particular grounded theory approach. CGT and SGT procedures are more clearly defined than those of CGGT in the seminal texts and may be more user‐friendly for a novice researcher (Cooney, 2010). In addition, SGT offers numerous tools to assist with data analysis, such as the coding paradigm to guide the construction of theory and the conditional/consequential matrix to elucidate broad contextual factors (Cooney, 2010; Howard‐Payne, 2016). However, the coding paradigm can also become cumbersome if it does not fit the data (Bryant & Charmaz, 2007a; Cooney, 2010; Evans, 2013). Some believe that CGGT and CGT methods are more flexible and offer greater creative freedom in theory development (Bryant & Charmaz, 2007c; Cooney, 2010; Evans, 2013). Another consideration is the availability of a grounded theory mentor to provide guidance about a particular approach (Artinian, 2009), especially if the study is part of graduate work. Through considering the study purpose, the philosophical implications of the different grounded theory approaches, and the specific pragmatic concerns of a situated study, researchers can determine the best fit for their work.

## 5 CONCLUSION

In the midst of continually evolving methodologies, it is crucial for researchers to be cognizant of the similarities and differences between the grounded theory approaches as they have significant implications for research procedures. This understanding facilitates methodological transparency (Amsteus, 2014) and philosophical/methodological congruence (Evans, 2013; McEwen & Wills, 2014; Ryan, 2018). Corbin and Strauss (2008) fittingly quoted Dewey (1934) regarding the grounded theory evolution, who observed, ‘If the artist does not perfect a new vision in his process of doing, he acts mechanically and repeats some old model fixed like a blueprint in his mind’ (p. 50). A thoughtful analysis of this evolution helps researchers to discriminate between the approaches to determine the best approach for their study and for who they are as researchers. Most important, this process will result in rigorous research that develops valuable knowledge to inform nursing practice.
